# Microbially competent 3D skin: a test system that reveals insight into host–microbe interactions and their potential toxicological impact

**DOI:** 10.1007/s00204-020-02841-z

**Published:** 2020-07-17

**Authors:** Lisa Lemoine, Ralf Dieckmann, Sascha Al Dahouk, Szilvia Vincze, Andreas Luch, Tewes Tralau

**Affiliations:** 1 Department of Chemical and Product Safety, German Federal Institute for Risk Assessment (BfR), Max-Dohrn-Strasse 8-10, 10589 Berlin, Germany; 2 Department of Biology, Chemistry, Pharmacy, Institute of Pharmacy, Freie Universität Berlin, Berlin, Germany; 3 Department of Biological Safety, German Federal Institute for Risk Assessment (BfR), Diedersdorfer Weg 1, 12277 Berlin, Germany; 4 Department of Food Safety, German Federal Institute for Risk Assessment (BfR), Max-Dohrn-Strasse 8-10, 10589 Berlin, Germany

**Keywords:** Microbial–skin tissue co-culture, Skin model, Commensals, Transcriptional changes, Method development

## Abstract

The skin`s microbiome is predominantly commensalic, harbouring a metabolic potential far exceeding that of its host. While there is clear evidence that bacteria-dependent metabolism of pollutants modulates the toxicity for the host there is still a lack of models for investigating causality of microbiome-associated pathophysiology or toxicity. We now report on a biologically characterised microbial–skin tissue co-culture that allows studying microbe–host interactions for extended periods of time in situ. The system is based on a commercially available 3D skin model. In a proof-of-concept, this model was colonised with single and mixed cultures of two selected skin commensals. Two different methods were used to quantify the bacteria on the surface of the skin models. While *Micrococcus luteus* established a stable microbial–skin tissue co-culture, *Pseudomonas oleovorans* maintained slow continuous growth over the 8-day cultivation period. A detailed skin transcriptome analysis showed bacterial colonisation leading to up to 3318 significant changes. Additionally, FACS, ELISA and Western blot analyses were carried out to analyse secretion of cytokines and growth factors. Changes found in colonised skin varied depending on the bacterial species used and comprised immunomodulatory functions, such as secretion of IL-1α/β, Il-6, antimicrobial peptides and increased gene transcription of IL-10 and TLR2. The colonisation also influenced the secretion of growth factors such as VFGFA and FGF2. Notably, many of these changes have already previously been associated with the presence of skin commensals. Concomitantly, the model gained first insights on the microbiome’s influence on skin xenobiotic metabolism (i.e., CYP1A1, CYP1B1 and CYP2D6) and olfactory receptor expression. The system provides urgently needed experimental access for assessing the toxicological impact of microbiome-associated xenobiotic metabolism in situ.

**Electronic supplementary material:**

The online version of this article (10.1007/s00204-020-02841-z) contains supplementary material, which is available to authorized users.

## Introduction

In terms of bacterial numbers and population density our skin comes second to the gut, harbouring more than 200 different bacterial genera with an overall population density between 1 million and 1 billion cells per cm^2^ (Grice et al. 2008, 2009;Ross et al. 2019). Set into relation this is a significant part of our microbiome, which as such not only outnumbers us cell- and genomewise, but also features a metabolic potential far exceeding our own in terms of biochemistry as well as genetic flexibility (Possemiers et al. 2011; Sender et al. 2016; Tralau et al. 2015; Yadav et al. 2018). While still in its relative infancy and thus maybe at time overstated our understanding of the microbiome’s impact on host biology continues to increase steadily. This is not the least thanks to massive parallel sequencing, metabolomics, metaproteomics and stable isotope probing-based approaches (Berry and Loy 2018; Isaac et al. 2019; Lamichhane et al. 2018; Turnbaugh et al. 2007). However, due to the limited choice of suitable in vitro models current studies mostly rely on analyses in situ, culture-independent approaches or, despite their restricted applicability, mouse models (Staley et al. 2017; Wang and Donovan 2015). The picture emerging from these studies shows an intricate relationship between the human host and its microbial colonists, the biological implications of which include commensalic functions such as vitamin and amino acid synthesis or immune modulation as was well as pathophysiologies such as allergies, chronic diseases, behavioural disorders or toxification of xenobiotics (Clemente et al. 2012; Platzek et al. 1999; Sowada et al. 2017; Tralau et al. 2015). The mechanisms underlying the latter are diverse and include substance-induced shifts of host microbiota composition, microbiome-catalysed chemical modifications or metabolism of xenobiotics as well as microbiome induced changes of the hosts gene expression (Collins and Patterson 2020). Traditionally, most research on microbial influence on xenobiotic metabolism has focused on the gut. Respective examples comprise more than thirty commercially available drugs, including blockbusters such as paracetamol (Clayton et al. 2009; Sousa et al. 2008; Tralau et al. 2015). At times harmless or merely affecting efficacy the corresponding consequences can also prove fatal. This has been tragically the case for sorivudine, highlighting the pressing need for a more systematic and better understanding of any potential microbial impact on toxification of drugs and chemicals (Sousa et al. 2008). Given the high metabolic potential of the various microbiomes and the fact that exposure to xenobiotics also occurs outside the gut it would be naïve, however, to restrict the search for such microbiome-associated substance-induced pathophysiologies to the gut. Indeed and albeit less well investigated in terms of potential microbiome interactions many active ingredients are, for example, also applied to skin.

Yet, detailed and systematic analysis of commensalic metabolism and its effects on host biology is often hampered as there is a lack of model systems able to emulate host–microbiome biology under controlled conditions, particularly if the aim is to move beyond the state of community analysis or measurement of basic metabolite patterns. With only a handful of systems available for the gut this is even more the case for skin. So far there is no microbially competent in vitro model commercially available that would allow skin-microbiome studies for extended periods of time.

Nevertheless, the biological relevance of such models is high and their potential applications extend well beyond toxicology. Modulation of skin inflammation, for example, is functionally dependent on commensals and the progress of inflammation depends, amongst others, on innate immune factors such as β-defensins and cathelicidin (Christensen and Bruggemann 2014; Gallo and Hooper 2012; Lai et al. 2010; Percoco et al. 2013). Released by keratinocytes in order to kill or inactivate pathogens (Lai and Gallo 2009), the expression of these antimicrobial peptides (AMPs) depends on commensal Toll-like receptor 2 (TLR2)-activation (Lai et al. 2010). Likewise, skin commensals have been shown to induce and control T cell responses in mouse models (Linehan et al. 2018; Schommer and Gallo 2013). Effects observed include increased production of pro-inflammatory molecules such as interferon-γ (INFγ) and interleukin (IL)-17A with the regulatory factors involved [IL-1α, IL-1β, IL-6, Transforming Growth Factor (TGF)-β] being partly induced or regulated by skin commensals (Feehley and Nagler 2014; Hasegawa et al. 2012; Naik et al. 2012; Veldhoen et al. 2008). These cytokines are also essential for the expression of various AMPs (Huang et al. 2002; Steinz et al. 2014). However, the mechanisms underlying the respective microbial immune-modulatory function are only partly understood. Particularly bacterially triggered modulations of toll-like receptor (TLR) responses extend the link into cellular signalling cascades beyond immediate immune reactions as the physiological implications of the IL-1R/TLR superfamily not only extend to inflammation regulation but also resistance to epithelial injury and epithelial homeostasis (Barton and Medzhitov 2003; Dunne and O’Neill 2003; Kubinak and Round 2012; Lopez-Castejon and Brough 2011).

Beyond extensively characterised immunomodulation dysbiosis of the skin’s microbiome has been associated with conditions such as atopic dermatitis or allergies and recent work highlighted the potential of skin commensals to form highly carcinogenic by-products from benzo[*a*]pyrene and other polycyclic aromatic hydrocarbons (Platzek et al. 1999; Sowada et al. 2014; Stingley et al. 2010). Yet, for many of the observed microbial dysbalances it still remains unclear if they are cause or rather consequence of the respective condition (Tralau et al. 2015). Even the presumably more straightforward hazard of carcinogenic metabolites remains challenging to assess, not the least due to the aforementioned lack of suitable model systems (Sowada et al. 2014, 2017). We now report on the development of a test system designed to study skin–microbe interactions in situ. The system relies on a commercially available 3D skin model, that is EpiDermFT™ from MatTek, which consists of epidermal and dermal layers, that are mitotically and metabolically active and exhibit in vivo-like morphological and growth characteristics which are uniform and highly reproducible. Moreover, the model was previously pre-validated for metabolically competent toxicity testing in vitro (Brinkmann et al. 2013; Hu et al. 2010) and genotoxicity testing using micronucleus and COMET assays (Pfuhler et al. 2014). In a proof-of-concept, this model has now been colonised using two previously isolated skin isolates, namely *Micrococcus luteus* 1B and *Pseudomonas oleovorans* 1C (Sowada et al. 2014). The selection of these organisms followed practical considerations and with the intended later application of studying potential microbiome-mediated substance toxification in mind. Both species are biologically relevant (Chiller et al. 2001; Wang et al. 2019), have an established potential for xenobiotic metabolism (Egea et al. 2017; Hanafy et al. 2016; Sowada et al. 2014; Viggor et al. 2020) and have been isolated repeatedly from healthy volunteers at different sites (Khayyira et al. 2020; Sowada et al. 2014; Steglinska et al. 2019; Wang et al. 2019). Amongst the skin’s Micrococcaceae *M.* *luteus* is the predominant species (Chiller et al. 2001). It is considered essential for the population balance of the skin’s microbiome (Epstein 2015) and usually accounts for 20–80% of the micrococci isolated (Davis 1996). Correspondingly *P.* *oleovorans* belongs to the Proteobacteria which make up to 34% of the whole skin microbiome (Kim et al. 2018). Lastly, both organisms bring the added bonus of being aerobes belonging to different Gram-categories which eases their laboratory handling.

The aim of this work was to develop a microbially competent skin model that provides access to microbe–host interactions and the toxicological impact of microbiome-mediated metabolism of xenobiotics under near in vivo conditions. Any such model has to be functional for at least 1 week in order to also pick up on slow or delayed xenobiotic modulations. Model functionality thus crucially depends on comparing its biology with what has been reported for commensalic skin interactions previously.

## Results

Two representative skin commensals were tested for stable colonisation of commercially available 3D skin models in a proof of concept study. Based on its performance in toxicological prevalidation studies the model of choice was EpiDermFT™ as distributed by MatTek (Brinkmann et al. 2013). The model was tested with two bacterial strains, the Gram-negative *P. oleovorans* and the Gram-positive *M. luteus*, both of which were previously isolated from healthy volunteers (Sowada et al. 2014).

### Establishment of stable microbial–skin tissue co-cultures

**Fig. 1:**
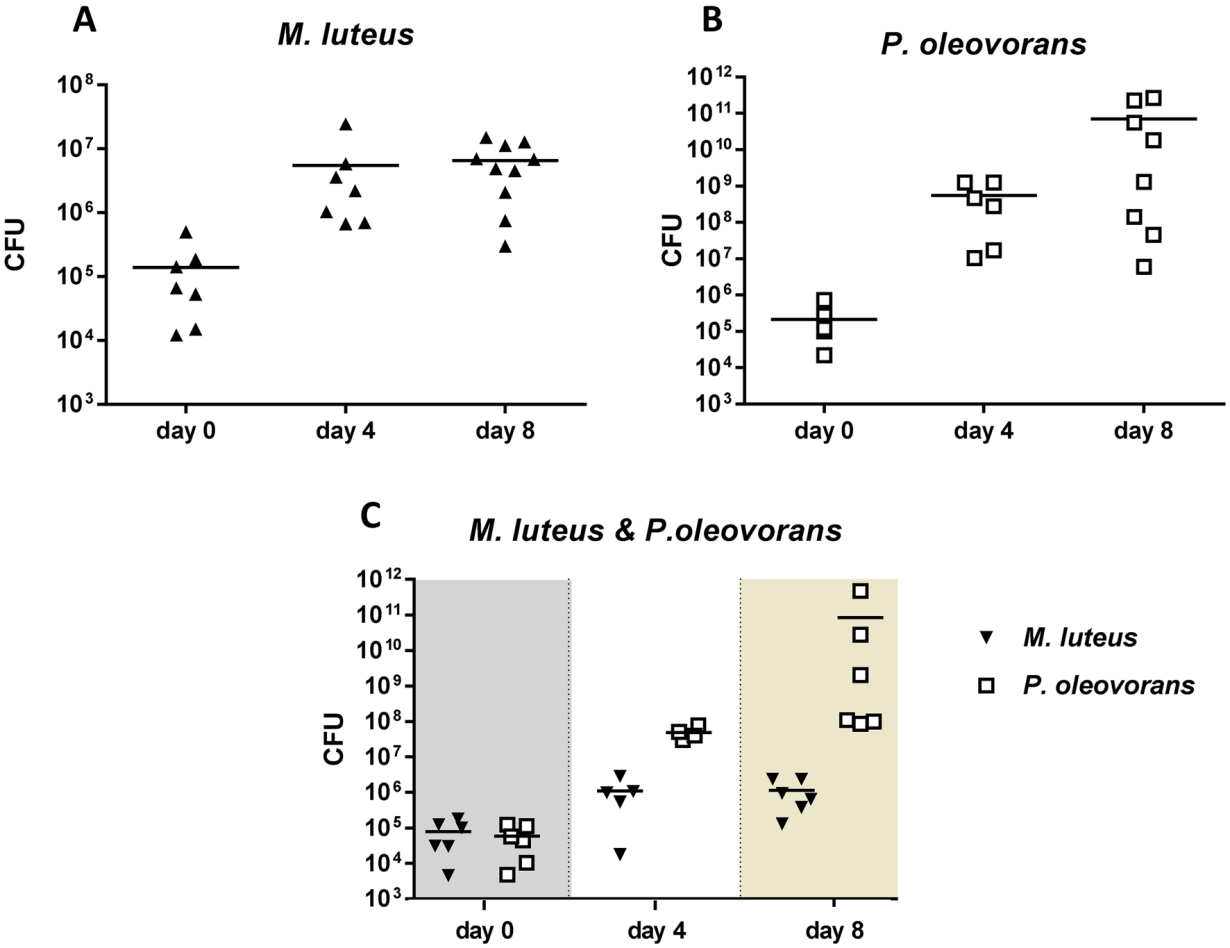
Plate counts from skin models on day 0, 4 and 8 of bacterial colonisation. The panels depict the results recorded for *Micrococcus luteus* (**a**), *Pseudomonas oleovorans* (**b**) and the mixed culture (**c**), respectively. Each point represents an independent experiment with the horizontal bars indicating the corresponding arithmetic mean. *CFU* colony-forming units

First tests focused on establishing microbial–skin tissue co-cultures using single strains or a mixed culture. Following bacterial inoculation the corresponding skin models were maintained over a period of 8 days with sampling performed on days 0, 4 and 8. Microbial–skin tissue co-culture formation and stability was followed by colony counts and strain-specific qPCR. Single cultures as well as the mixed culture viable colony counts (CFU/cm^2^) showed microbial–skin tissue co-culture establishment to occur during the first 4 days with bacterial cell numbers of *M. luteus* remaining largely stable thereafter (Fig. 1a). However, in both culture scenarios (single vs. mixed culture) *P.* *oleovorans* repeatedly reached higher cell numbers. In the mixed culture this resulted in *P.* *oleovorans* outcompeting *M. luteus,* the latter growing in lower numbers than when cultured alone (Fig. 1b, c). Strain-specific real-time quantitative PCR (data not shown) confirmed this data.

Microscopy and Gram-staining was concomitantly used to visualise bacterial colonisation in situ. The respective slides showed *M. luteus* to exclusively stay on the surface of the skin, whereas *P. oleovorans* appears partially to penetrate into the (epi)dermal layers (Supplementary Fig. S3a). Moreover, and in line with the CFU-data the slides visually confirmed *P.* *oleovorans* to outnumber *M. luteus* in microbial–skin tissue co-culture (Supplementary Fig. S3b). None of the recordings indicated any cross-contamination or foreign contamination with microorganisms. This was also confirmed by supplementary 16S-PCR and sequencing.

### Impact of bacterial co-colonisation on skin model biology

**Fig. 2:**
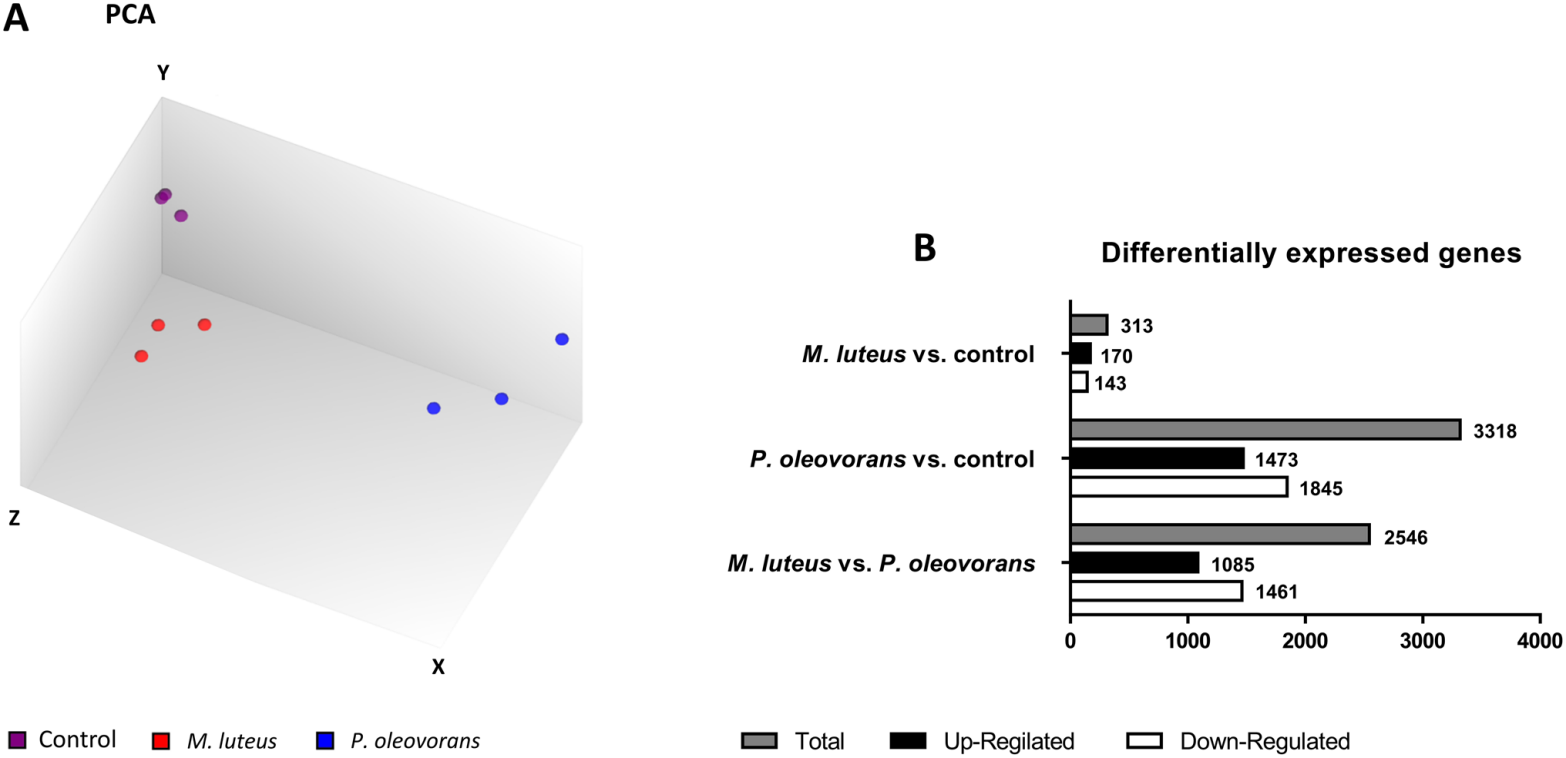
Transcriptional response of EpiDermFT™ models on day 8 of microbial colonisation. Mapping 81.4% of the available transcripts PCA shows clear separation of the untreated control from tissues colonised with *M. luteus* or *P. oleovorans* (**a**). Number of differentially expressed genes in skin colonised with *M. luteus*, *P. oleovorans* and the mixed culture (**b**). All experiments were conducted in triplicate

Next the impact of microbial co-colonisation on the skin models was assessed. For this purpose, Human Clariom™ S assays were subsequently used to record transcriptomic profiles of models colonised with *M. luteus* or *P.* *oleovorans*. The assay provides a transcriptomic snapshot of a core-set of 20,000 well-annotated genes, thus allowing a basic functional assessment of the skin’s global gene expression and molecular network interactions prior and after microbial colonisation. Co-colonisation had a clear effect on the skin with principal component analysis differentiating the untreated controls from the microbially competent models (Fig. 2a). In total 313 and 3318 transcripts were found to be specifically affected by the presence of *M.* *luteus* and *P.* *oleovorans*, respectively (Fig. 2b).

**Fig. 3:**
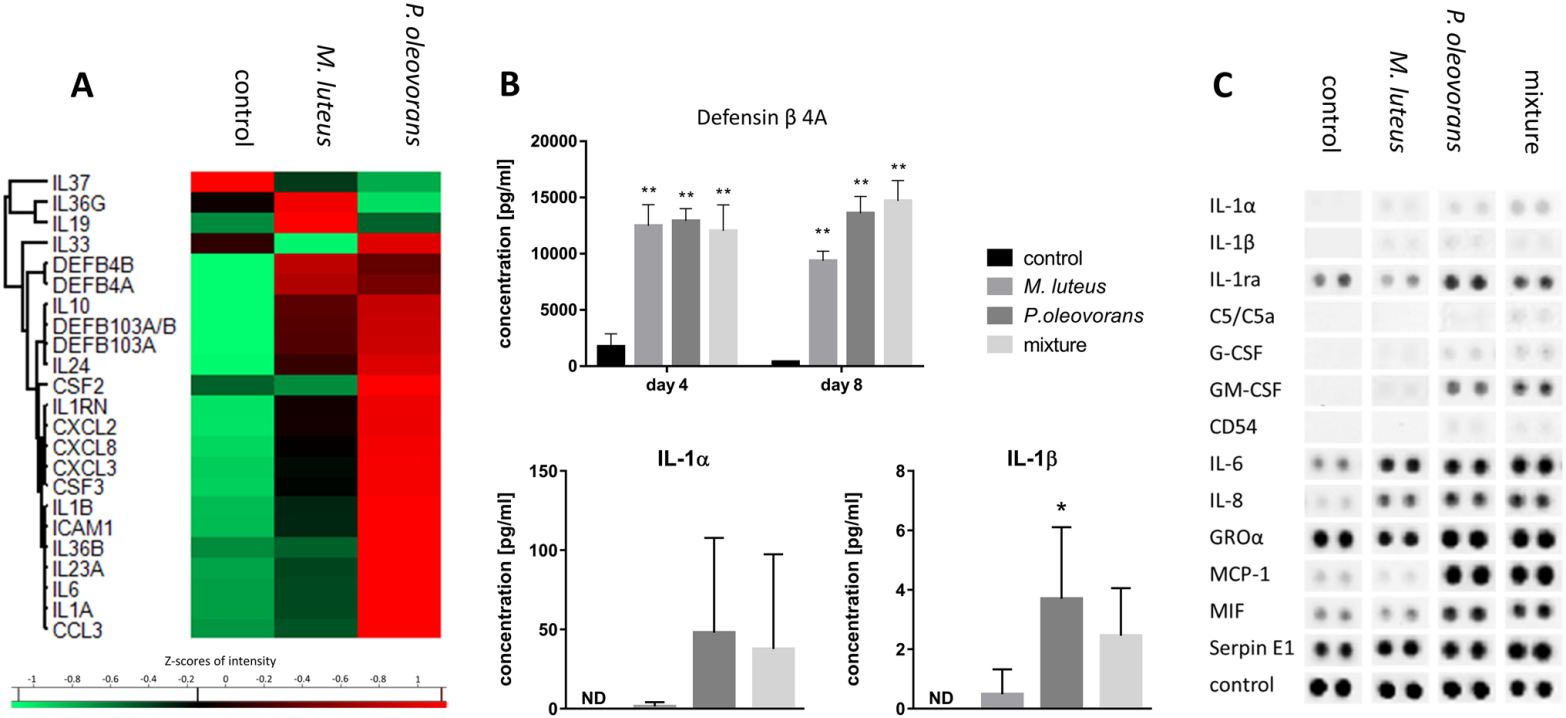
Expression of various cytokines and defensins in microbially competent skin models. The cluster map records the transcriptional state at day 8 of microbial colonisation as indicated (**a**). Shown are the gene symbols and Z-scores of significantly differentially expressed genes with an *F* value < 0.05 across at least three independent experiments. Concomitant excretion of defensin β 4A and IL-1α/β into the supernatant was quantified using an ELISA and FACS analysis, respectively (**b**). Shown are mean concentrations with error bars indicating standard deviation. All values are significant within *p < 0.05 or **p < 0.01, values labelled “ND” were not detectable. Similarly, cytokine excretion into the supernatant was verified qualitatively using a proteome profiler array (**c**)

Preliminary functional IPA analysis indicated reduced cell death and moderate effects on cell growth, proliferation and tissue development, including vasculogenesis (Supplementary Fig. S4). Although the underlying transcriptional changes occurred with both strains, the effectual tendencies were more pronounced with *P. oleovorans*. In depth analysis showed most of the affected transcripts to relate to immune functions with the AMPs defensin β 4A and B being amongst the most differentially regulated genes (Fig. 3a). Befittingly increased levels of defensin β 4A were also detected in the culture supernatant (Fig. 3b). Other AMPs such as Cathelicidin-related antimicrobial peptides (CAMP) or RNase7 were not found to be differentially expressed. Nevertheless, IPA analysis showed high activity of CAMP-regulated transcripts (*p* = 6.73 × 10^−10^), indicating some activation of the corresponding molecular pathways.

The co-colonised models generally featured increased transcription of *TLR2* with *P. oleovorans* also inducing *TLR6*. Expression of *TLR3* was repressed though and while the stress- and immune-responsive NF-κB pathway clearly reacted differently to *M. luteus* or *P. oleovorans* it nevertheless failed to provide any clear functional response on transcriptional level (Supplementary Fig. S5a).

Taken together the transcriptional responses of the co-colonised models thus indicate a state of increased immune competence. This was further confirmed by the expression patterns of various pro- and anti-inflammatory cytokines including IL1-α, IL1-β, IL-10 and TGF-β. Again, the observed cytokine patterns showed some strain-specific induction, particularly for *P. oleovorans* (Fig. 3a). Protein secretion was in line with the gene expression data except for Macrophage migration inhibitory factor (MIF) and Monocyte chemoattractant protein **(**MCP)-1, both of which were recorded at elevated levels in the presence of *P. oleovorans* but failed to show matching transcriptional induction (Fig. 3a–c). Interestingly, exposure to *P. oleovorans* also induced partial expression of signalling pathways of triggering receptors expressed on myeloid cells (TREMs) (Supplementary Fig. S5b and c). Amongst other functions these receptors act as important immune modulators and are known to be activated by commensal and pathogenic bacteria (Varanat et al. 2017; Wu et al. 2011). Overall, the transcriptional response of combined pro- and anti-inflammatory effects mirrors the reaction against commensals in vivo (Kubinak and Round 2012; Meisel et al. 2018; Nutsch and Hsieh 2012).

**Fig. 4:**
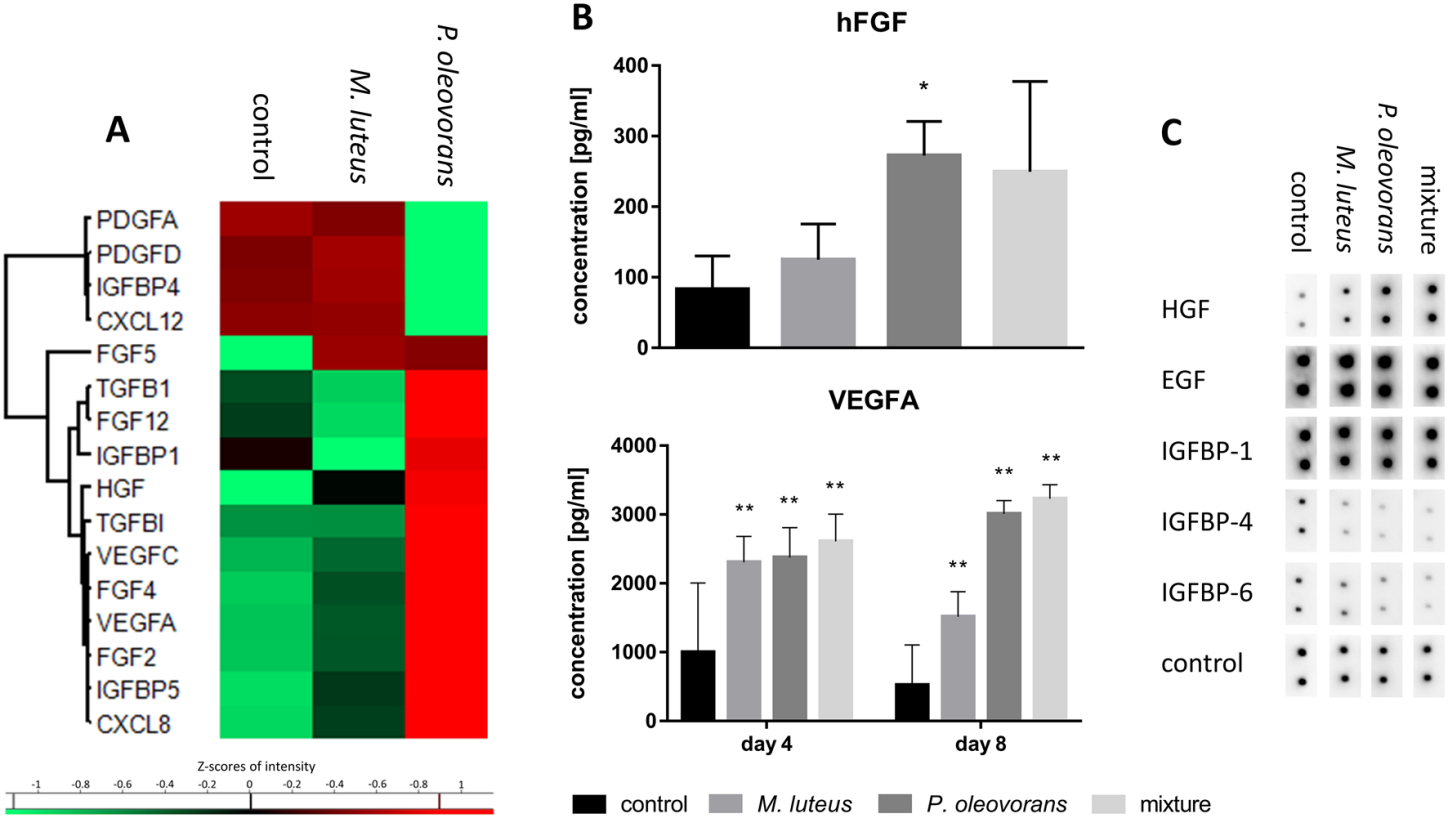
Expression of cellular growth factors in microbially competent skin models, colonised as indicated. The cluster map records the transcriptional state at day 8 of microbial colonisation (**a**). Shown are the gene symbols and Z-scores of significantly differentially expressed genes with an *F* value < 0.05 across at least three independent experiments. Concomitant excretion of hFGF and VEGFA into the supernatant was quantified using an ELISA (**b)**. Shown are mean concentrations with error bars indicating standard deviation. All values are significant within *p < 0.05 or **p < 0.01. Expression of growth factors into the supernatant was further verified qualitatively using a proteome profiler array (**c**)

Apart from triggering increased immune competence microbial co-colonisation also had marked transcriptional effects on many genes of the fibroblast growth factor family (FGF2, 4, 5 and 12), the vascular endothelial growth factor family (VFGFA and VFGFC), the transforming growth factor beta family (TGFB1 and TGFBI), and the insulin-like growth factor-binding protein family (IGFBP1, 3, 2 and 5) (Fig. 4a). Again, some of these effects were rather strain specific with *P. oleovorans* tending to produce a more pronounced response. Altogether the transcriptional effects seemed to be representative of the model’s priming towards more fine-tuned differentiation rather than indicating functional differentiation as such, for effects on protein expression were far less pronounced (Fig. 4b, c). The latter only related well for selected transcripts such as the microbially induced expression of VFGFA and hepatocyte growth factor (HGF), or the downregulation of IGFBP6 and IGFBP4, respectively.

Commensal communities are also known to induce strong differential expression of many olfactory receptors (ORs). With about 400 members this receptor family is expressed throughout the body, regulating physiological cell functions well beyond olfaction. In skin this includes keratinocyte proliferation, migration, and re-epithelialisation of keratinocytes (Cheret et al. 2018; Denda 2014). Markedly microbial–skin tissue co-culture with *P. oleovorans* or *M. luteus* led to 77 or 15 differentially regulated ORs (Supplementary Fig. S6), with the functional implications remaining unclear though.

### Impact on metabolic competence

**Fig. 5:**
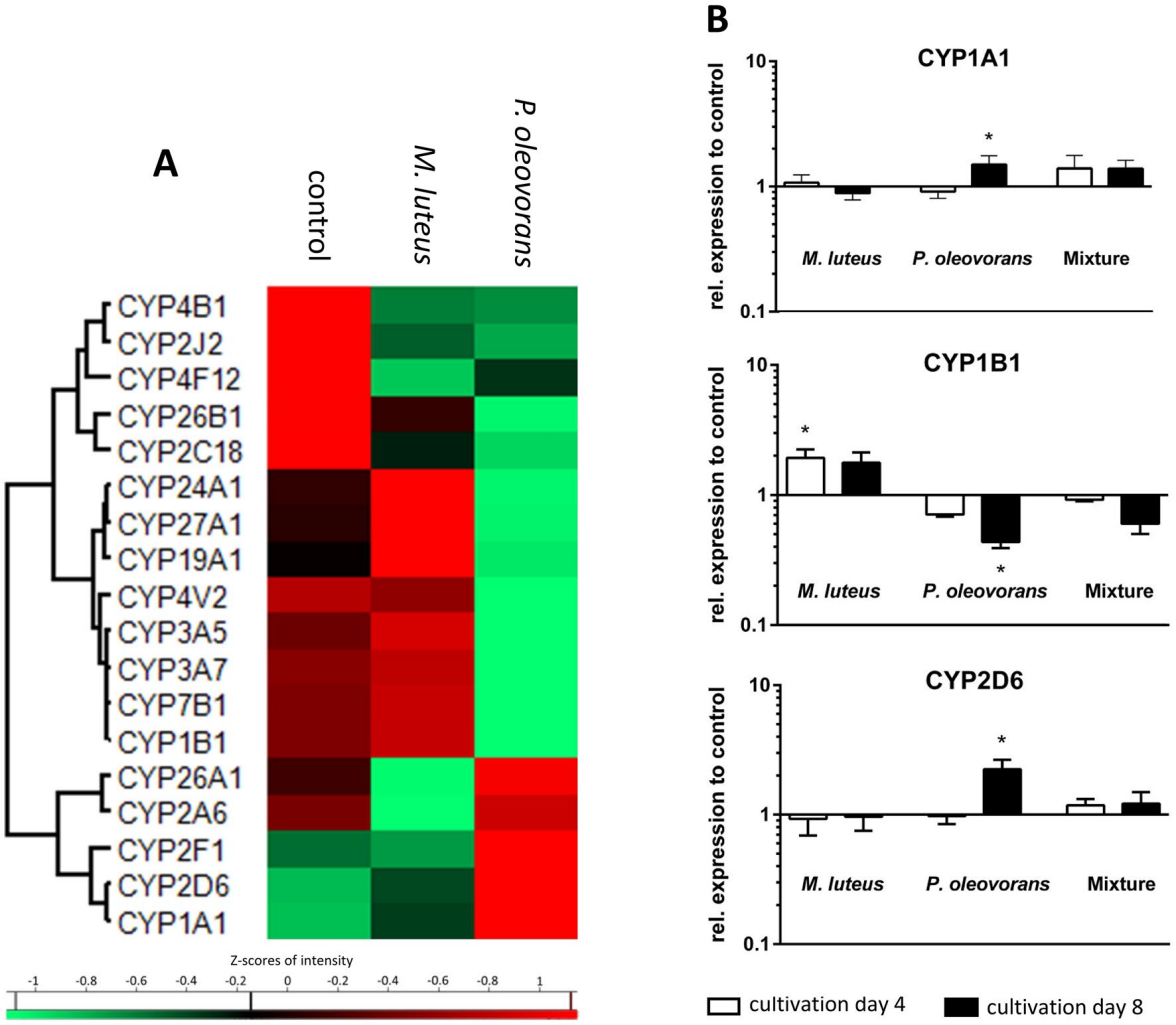
Expression of various cytochrome P450-dependent monooxygenases (CYPs) involved in phase I metabolism in the skin with the cluster map recording the transcriptional state at day 8 of microbial colonisation (**a**). Shown are the gene symbols and Z-scores of significantly differentially expressed genes with an *F* value < 0.05 across at least three independent experiments. Expression of key transcripts was quantified further using RT-PCR (**b**)

In light of the intended use for toxicological studies the colonised skin models were further analysed with respect to transcription of various cytochrome P450-dependent monooxygenases (CYPs), an enzyme class that represent the mainstay in phase I metabolism (Fig. 5a, b). Of the 63 CYPs examined almost 30% were transcriptionally affected in microbial–skin tissue co-culture, with most of the responses being strain specific to various degrees. However, amongst the six CYPs characteristic for skin metabolism (i.e., CYP1A1, CYP1B1, CYP2B6, CYP2E1, CYP2D6, and CYP3A) only CYP1A1 and CYP2D6 were subject to some additional induction in the presence of *P. oleovorans*, with transcript levels rising 1.7-fold and 2.4-fold compared to the uncolonised control. This trend was also confirmed by quantitative RT-PCR. Taken together the results show that while co-colonisation adds an additional layer of microbial metabolism with potentially biasing effects on the skin’s inherent CYP-mediated phase I capacity, its impact is likely to remain moderate.

## Discussion

The human microbiome is an integral part of our (patho)physiology. Yet, an overwhelming part of microbiome research still concentrates on the description of microbial communities and their population dynamics (Round and Palm 2018; Sazal et al. 2020; Walter et al. 2020). Contrastingly and with hardly any in vitro systems available studying the underlying mechanisms and causality of host–microbe interactions remains a major challenge. For the gut Human Microbiota-Associated Mice (HMA) often continue to be a preferred system, despite all technical limitations and ethical issues. With only 15% of gut bacterial lineages shared compositional differences of the intestinal microbiomes in man and mice far exceed those within species (Ley et al. 2005) and consequently humanised systems tend to suffer from poor long-term stability (Rawls et al. 2006). Alternatively, bioreactors have hence been used as a simple and effective method to study microbial metabolism and community dynamics under various dietary and xenobiotic influences (Guzman-Rodriguez et al. 2018). Yet, as with other techniques these systems are also subject to limitations such as culturability, long-term stability and lack of direct intestinal interaction (Guzman-Rodriguez et al. 2018; Vrancken et al. 2019). The problem becomes even more evident when moving to commensal communities outside the gut. Rodent skin for example not only features its own species-specific microbiome, but also is profoundly different in terms of physiology and metabolism (Gerber Peter et al. 2014; Oesch et al. 2014).

For toxicological and mechanistic studies of the skin, human 3D skin models have hence long been tools of choice, albeit lacking any microbial competence in situ (Rademacher et al. 2018). Systems used to study the pathophysiology of skin–microbe interactions (i.e., during infectious or inflammatory settings) comprise cell cultures with added bacterial endotoxins (Kampfer et al. 2017; Lai et al. 2009), two-dimensional cell cultures in the presence of live pathogens or the odd three-dimensional approach with bacteria added to the culture medium (Barrila et al. 2017; Mason et al. 1998; Mohiti-Asli et al. 2014). Additionally, there is a stratum corneum model from van der Krieken et al. (2016), where bacteria are applied to the apical surface of dead corneocytes or a model from de Breij et al. (2012), where an epidermal skin equivalent is colonised by *Acinetobacter* species. While the latter models come close to the in vivo situation, at least structurally, they nevertheless lack the option to study microbial interaction with living keratinocytes and fibroblasts. There are also studies on colonising 3D skin equivalents which feature dermis, epidermis and a fully differentiated stratum corneum as well as surface contact with ambient air. However, these models have not been pre-validated for toxicity studies. Also, the colonisation in these studies has been limited to 48–120 h. Yet, the studies of Sowada et al. (2017) show that at least for PAHs microbe-mediated toxicity occurs predominantly at time-points later than 5 days. Moreover, the aforementioned models often lack a detailed biochemical characterisation (Bojar 2015; Cadau et al. 2017; Holland et al. 2008; Maboni et al. 2017; Popov et al. 2014).

For our studies we used MatTek’s full thickness skin model EpiDermFT™. Together with fully functional barrier properties and a metabolic complexity close to that of in vivo skin this model has a proven track record as reliable testing system for skin toxicity (Black et al. 2010; Brinkmann et al. 2013; Wills et al. 2016). The successful microbial colonisation reported in this study is a major functional amendment as it provides, to the best of our knowledge, the first testing system for assessing “longer term” skin-microbiome interactions in situ. The skin models were efficiently colonised with commensal communities of *M.* *luteus* and *P.* *oleovorans*, with the letter performing slightly better than the first. Following inoculation microbial–skin tissue co-cultures took up to 4 days to establish and remained stable at least until day 8 with colony counts similar to what is reported for skin in vivo (up to 10^9^ CFU/cm^2^) (Ross et al. 2019; Tralau et al. 2015). In general, *P.* *oleovorans* was repeatedly observed to grow to higher cell counts compared to *M.* *luteus* both in single and mixed microbial–skin tissue co-culture. For some experiments this resulted in cell counts of up to 10^11^ cells/cm^2^ for *P.* *oleovorans* on day 8. This is possibly due to an increased robustness against nutrient limitation and this organism’s ability to colonise not only the stratum corneum but to penetrate into the epidermis and dermis, particularly in absence of a fully functional immune response. This growth behaviour matches that reported for other *Pseudomonas* spp. in human skin biopsies (Nakatsuji et al. 2013). In contrast, *M.* *luteus* cell counts were slightly reduced in mixed microbial–skin tissue co-cultures probably due to its dependence on a more restricted range of carbon sources (Young et al. 2010). What is more, is that 3D skin models such as EpiDermFT™ do not have sweat glands or hair follicles. This limits the choice of naturally available nutrients to peptides and lipids, restricting access to urea, ammonia, vitamins or sugars (Scharschmidt and Fischbach 2013). However, the latter will at least partially be compensated for by diffusion of glucose from the cell culture medium into the dermal and epidermal layers of the model (Khalil et al. 2006; Ullah et al. 2018). This provides some selectional advantage for the more invasive *P.* *oleovorans* as even a thin stratum corneum layer constitutes a considerable barrier for passive diffusion (Ullah et al. 2018).

Transcriptional influence of the skin microbiome on its host is well established, although little understood and thus of high interest (Duckney et al. 2013; Linehan et al. 2018; Ridaura et al. 2018; Wanke et al. 2011). The extent and impact are community specific, something also seen in our models where *P.* *oleovorans* influenced the transcription of nearly 3300 genes. That is nearly tenfold the number of transcripts affected by *M.* *luteus* and in the same order of magnitude what is seen in germ-free mice in response to microbial colonisation (Meisel et al. 2018). Part of this gene response is likely to be the consequence of *P.* *oleovorans* penetrating the epidermal barrier and its relatively high cell count of up to 10^11^ CFU/cm^2^ on the eighth day of cultivation. With *P. oleovorans* being an opportunistic pathogen an immunogenic response is inherently necessary to maintain a healthy commensal community. Duckney et al. (2013) reported a strong and predominantly inflammatory response in skin models when mimicking complete barrier breakdown. The effects observed in our colonised models are less acute and rather in line with what is seen for commensal communities in vivo. For example, similar to what is seen in host–microbiome animal studies both organisms lead to increased gene expression of β-defensins DEFB3A and DEFB4A (Lai et al. 2010; Rademacher et al. 2019). These β-defensins are critical but not exclusive for host defence, with induction of *DEFB3A* relying, amongst other things, on activation of TLR2 (Shin and Choi 2010). Befittingly we also see increased levels of *TLR2* expression in the skin subsequent to microbial colonisation. The increase was significantly more pronounced in skin models colonised with *P.* *oleovorans*, together with a slight but significant upregulation of TLR 6. Induction of TLR2 by LPS from *Pseudomonas* species has been shown earlier (Shin et al. 2013). Homo- or heterodimers of TLR2 or TLR2/TLR6 are capable of discriminating various lipopeptides (Takai et al. 2014) and are involved in immune modulation, including elicitation of cytokine secretion and promotion of regulatory T (Treg) cell (Nawijn et al. 2013; Netea et al. 2008; van Maren et al. 2008) and Th17 cell responses (DePaolo et al. 2012; Reynolds et al. 2010; Xu et al. 2017; Zhao et al. 2015). Results from preliminary screens with THP-1 cells further support this as exposure to medium from microbially competent models leads to secretion of IL-23, a cytokine crucial for Th17 development and adaptive immunity (Supplementary Fig. S7) (McGeachy et al. 2009; Zielinski et al. 2012). Various TLRs have been implicated to modulate the immune responses to commensals in vitro (Kubinak and Round 2012; Maier et al. 2018; Ren et al. 2016) as well as in vivo (Oppong et al. 2013; Round et al. 2011), often promoting mutually beneficial microbe–host interactions. Amongst the respective target cytokines are IL-1α, IL-1β, IL-6 (Hasegawa et al. 2012; Naik et al. 2012; Ren et al. 2016) and IL-10 (Cosseau et al. 2008; Jun et al. 2017; Neish 2009), all of which we found to be elevated in the presence of *P.* *oleovorans* and *M.* *luteus*. The transcription of these cytokines is partly subject to TREM-1 signalling, which was significantly induced in models colonised with *P.* *oleovorans* (Lagler et al. 2009; Rai and Agrawal 2017; Tessarz and Cerwenka 2008). Commensalic influence on IL-1 signalling is known to modulate effector T cell responses. The corresponding modulation and fine-tuning of immune responses is essential for a functioning host–microbiome relationship as well as for maintaining a healthy skin (Naik et al. 2012; Park and Lee 2017). In fact, maturation of IL-1α and IL-1β crucially depends on commensal presence (Lopez-Castejon and Brough 2011; Naik et al. 2012). Indeed microbial–skin tissue co-cultures with both organisms showed an increase in gene expression and excretion for both cytokines. Other genes of the interleukin-1 family were also upregulated, including IL-33 and IL-36β for *P. oleovorans* and IL-36γ for *M. luteus*. Similarly the observed expression of anti-inflammatory messengers such as IL-10 and TGF-β has been linked to commensal colonisation and associated immune tolerance (Fung et al. 2016; Meisel et al. 2018; Ueda et al. 2010). Altogether the response of the co-colonised models thus is very much similar to what is observed in vivo, with increased immunomodulation and immunotolerance, respectively (Nutsch and Hsieh 2012). Similarly, many of the aforementioned transcription factors and genes found to be transcriptionally affected by co-colonisation match those seen in skin development and differentiation in vivo. Examples include the host angiogenesis transcription factor Ephrin-A1 as well as the growth factors FGF2, HGF, PTGER4, VEGFA and VEGFC or ORs such as OR2A7 (Linehan et al. 2018; Sajib et al. 2018; Stappenbeck et al. 2002). The latter is known to play an important role in various processes of the skin, including keratinocyte proliferation (Tsai et al. 2017). The regulation of ORs by human commensals has so far been little studied. Pluznick et al. (2013) showed critical involvement of OR51E2 in intestinal host–microbiome signalling. In our skin models this receptor was upregulated in the presence of *P.* *oleovorans*. Interestingly, seven of the genes from the OR family were differentially regulated in both microbial–skin tissue co-cultures. Continuatively, it seems worthwhile to assess the general role of ORs in skin-microbiome signalling in more detail.

Colonisation of the skin models also had a marked influence on CYP expression with almost 30% of CYPs affected, including the toxicologically relevant CYP1A1. In mice CYP1A1-facilitated detoxification of carcinogens has been implicated to rely on TLR2-dependent signalling (Do et al. 2012). Therefore, the differential expression of TLR2 with *P.* *oleovorans* is likely also causal for this organism’s marked induction of CYP1A1. While little is known for skin, the overall microbial influence on CYP expression in the gut has been reported previously. This includes CYP3A, CYP2C9, CYP1A and CYP2D6, for which microbial metabolites can either serve as substrates, inductors or inhibitors (Claus et al. 2011; Tralau et al. 2015). Biological pathway analysis also implicated microbial influence of both organisms on other key processes such as proliferation, keratinocyte differentiation and apoptosis. In general, *P.* *oleovorans* seems to have a greater influence on processes pertained to cellular movement of the dermis (fibroblasts and connective tissue), whereas *M.* *luteus* rather influences keratinocyte movement. This is probably due to the different habitats of the organisms. So far, little is known about the influence of microorganisms on cell proliferation and differentiation in the skin. However, our results confirm that the balance between epidermal proliferation and differentiation is altered in response to microbial colonisation (Meisel et al. 2018). The underlying pathways involve signalling of MAPK and NF-κB as well as IL-1 and DEFB3/4A, all of which we found to be affected (Eller et al. 1995; Nishimura et al. 2004; Preciado et al. 2005).

Currently, the model is limited to a selected number of species as it has not been tested for entire swabs or community stamps. As such it will also always be limited to the cultivation bias inherited by all growth-based models. This is not the least because it carries the inherent limitations of MatTek’s EpiDermFT™ such as the absence of sweat glands and selections glands, both of which contribute to the nutrient pool of natural skin communities. However, the microbially mediated changes in the skin matched what is known from other skin studies and mouse models. This is true for the observed alterations in gene expression such as the IL-1 family, the secretion of antimicrobial peptides and the predicted influence on skin differentiation and proliferation. As an extendable microbial–skin tissue co-culture system this model hence provides a good system for studying selected skin host–microbiome interactions including microbiome-mediated substance toxification in situ over extended periods of time.

## Materials and methods

### Chemicals and media

If not mentioned otherwise chemicals were purchased at purities greater than 98% from Sigma-Aldrich (Taufkirchen, Germany) or Carl Roth (Karlsruhe, Germany), respectively. Media for the 3D skin models were sourced from MatTek (Ashland, MA) while molecular reagents and kits were routinely obtained from Qiagen (Hilden, Germany) and Invitek (STRATEC Molecular GmbH, Berlin, Germany). Primers were purchased from Metabion (Martinsried, Germany).

### Bacterial isolates and bacterial growth

Microbial–skin tissue co-cultures were set up using two previously enriched skin-commensals, that is *Micrococcus luteus* 1B and *Pseudomonas oleovorans* 1C (Sowada et al. 2014). When not applied on the surface of the skin models bacteria were routinely grown as shake flask cultures in lysogeny broth (LB) at 200 rpm and 32 °C. Growth was routinely monitored using optical density (OD, *λ* = 600 nm) and correlated to colony-forming units (CFU) as established by serial plate counts (PC) as required. Cells used for skin model inoculation were harvested at an OD_600_ of 0.4–0.9 with 7.500 g for 8 min and washed once in PBS (MatTek, Ashland, MA, USA). The pellet was subsequently re-dissolved in 15 µl PBS and 10^4^–10^6^ cells used for skin inoculation.

### Whole-genome sequencing (WGS)

For each isolate a single colony grown on LB agar was inoculated in liquid LB and cultivated under shaking conditions at 150  rpm and 37 °C for 22 ± 2  h. Subsequent extraction of DNA was performed using the PureLink^®^ Genomic DNA Mini Kit (Invitrogen, Carlsbad, CA, USA). Sequencing libraries were then prepared with the Nextera XT DNA Sample Preparation Kit (Illumina, San Diego, CA, USA) according to the manufacturer’s protocol. Paired-end sequencing performed in 2 × 301 cycles on an Illumina MiSeq benchtop using the MiSeq Reagent v3 600-cycle Kit (Illumina).

### Tissue culture

Skin models (EpiDermFT™) were obtained from MatTek (Ashland, MA, USA). Three days before shipment the models are started to cultivated in antibiotic-free medium. Upon arrival the models were directly transferred into six-well plates (Greiner Bio-One, Frickenhausen, Germany) and allowed to recover overnight in 2.5 ml of antibiotic-free EPI-100-MM-ABF at 37 °C and 5% CO_2_ as recommended by the manufacturer. Following recovery the models were then subjected to bacterial inoculation or solvent treatment, respectively. Models were subsequently maintained at 37 °C in a humidified atmosphere of 5% CO_2_ for up to 8 days with culture media being exchanged daily, following the recommendations of the manufacturer who warrants culture stability for up to 2 weeks. Tissues and media not used upon completion were harvested, shock-frozen in liquid N_2_ and stored at − 80 °C as appropriate. Model sections to be used for bacterial staining were transferred into embedding medium prior to freezing.

The medium of microbial–skin tissue co-culture models was checked daily for contamination by sampling for possible bacterial growth by OD_600_-measurements and plating. Follow-up experiments were only carried out if the medium was free of contamination.

Please note that it is highly recommended to use different plates for different microbial–skin tissue co-cultures. This minimises the risk of cross-contamination. Also, the volume of the bacterial inoculum applied topically should not exceed 15 µl.

### Bacterial quantification: skin models

Bacterial growth in microbial–skin tissue co-culture was quantified using PC or strain-specific quantitative PCR (ss-qPCR) (as described in the supplementary section Method S1), respectively. For the PC bacterial imprints were obtained from the surface of the skin models using 2 cm^2^ of velvet cloth. The cloth was soaked in sterile PBS and applied with gentle pressure to the surface of the skin model before being subsequently transferred into 1 ml of sterile PBS. Following incubation in a thermomixer (Eppendorf, Hamburg, Germany) at room temperature for 30 min at 6000 rpm the velvet was wrung out and 100 µl of the bacterial PBS-suspension were used to set up serial dilutions on LB agar. After 24 h at 37 °C bacterial counts were then recorded as CFU/ml.

In order to preclude the possibility of a contamination with other microorganisms, we have performed a PCR with 16S-rRNA gene-specific primers (Tralau et al. 2011) with the DNA of the individual models, followed by sequencing of the PCR products at Eurofins (Ebersberg, Germany).

### Bacterial staining of co-colonised skin models

For bacterial stains frozen model sections (~ 1 cm^2^) were cut in a cryomicrotome at − 20 °C. Slices were set to measure 5 μm in diameter and then subjected to standard Gram-staining using the Gram stain tissue kit (Sigma, St. Louis, MO, USA). Staining was performed according to the manufacturer’s instructions relying on precooled acetone (− 20 °C, 20 min) as fixation agent. The results were recorded using a standard Axio Observer A1 microscope (Zeiss, Oberkochen, Germany).

### THP-1 cell culture

THP-1 cells were obtained from Leibniz Institute DSMZ—German Collection of Microorganisms and Cell Cultures (Braunschweig, Germany). Growth was routinely performed using RPMI 1640 medium (PAN-Biotec, Aidenbach, Germany) supplemented with 10% (v/v) FBS (Biochrom, Berlin, Germany), HEPES; 10 mM, l-glutamine (2 mM), sodium pyruvate (1 mM) and penicillin/streptomycin (100 U/ml) (PAN-Biotec). For routine cell culture cells were seeded at 1 × 10^5^ cells per ml into T75 flasks at 37 °C, 5% CO_2_ and 95% humidity and passaged every 3–4 days.

Cells used for cytokine arrays were seeded into 96-well plates at a density of 0.5 × 10^6^ cells per 96 well plate and left to rest for 24 h before being subjected to treatment with supernatants from the respective skin models for another 24 h.

### mRNA analysis

Total RNA was recovered subsequent to cell harvesting with a TissueLyser II (Qiagen, Hilden, Germany) using a TRIzol-based protocol (Chomczynski and Sacchi 1987). Briefly, following cellular disruption at 20 Hz for 3 min, total RNA was extracted using TRIzol™ Reagent (Invitrogen) according to the manufacturer’s instructions. The RNA-integrity (RIN) was analysed with an Agilent 2100 Bioanalyzer System (Agilent Technologies, Waldbronn, Germany) and the Agilent RNA 6000 Nano Kit (Agilent Technologies, Waldbronn, Germany) as described by the manufacturer. Following quality assessment samples were either stored at − 80 °C or directly used for quantitative RT-PCR or microarray analysis, respectively.

Microarray analysis was performed using triplicate Human Clariom™ S assays (Applied Biosystems, Foster City, CA, USA) at ATLAS Biolabs (Berlin, Germany). The corresponding RNA-samples all featured a RIN-score > 7. Subsequent data evaluation and interpretation was then carried out in house, relying on the Transcriptome Analysis Console 4.0.1.36 (TAC) (Applied Biosystems, Foster City, CA, USA) (± 2 fold-change; *p* < 0.05) and Ingenuity Pathway Analysis (IPA) (QIAGEN Inc., https://www.qiagenbioinformatics.com/ products/ingenuity-pathway-analysis, Qiagen, Hilden, Germany) software packages. The latter was used with its core analysis module using ± 1.5-fold-change and *p* < 0.05 as cut-off values.

Concomitant analysis of gene-specific expression was performed using quantitative RT-PCR. In brief 500 ng of mRNA were reversely transcribed using oligo-dT primers and the Omniscript^®^ Reverse Transcription Kit (Qiagen, Hilden, Germany). Subsequent amplification and detection of transcript levels relied on gene-specific primers (Supplementary Table S2) together with Fast SYBR^®^ Green Master Mix (Applied Biosystems, Thermo Fisher Scientific, Darmstadt, Germany) as instructed by the manufacturer. All experiments were carried out in triplicate with *GAPDH* as house-keeping control and using a 7500 Fast Real-time cycler by Applied Biosystems (Thermo Fisher Scientific, Darmstadt, Germany). Relative transcript levels were calculated based on *c*_T_-values using the 7500 Fast SDS Software.

### Quantification of secreted factors

Cytokine secretion of microbially competent skin and THP-1 cells was measured using a Proteome Profiler™ Human Cytokine Array Panel A (R&R Systems, Abingdon, UK). Il-1α and β were additionally quantified in microbial–skin tissue co-culture using a custom human 7-plex panel (Biolegend, London, UK) and FACS. Growth factor secretion was measured using the Human Growth Factor Array C1 (RayBiotech, Peachtree Corners, GA, USA). Levels of VEGFA and Defensin β4A or hFGF were determined with ELISA kits from RayBiotech, Inc. (Norcross, GA, USA) or USCN Life Science (Wuhan, China) according to the manufacturer’s protocol, respectively.

### Western blot

Expression of selected proteins in skin models was verified using Western blotting. For protein extraction skin tissues in PBS (250 µl) were lysed with a TissueLyser II (Qiagen, Hilden, Germany) operated for 5 min at 20 Hz in in presence of Protease Inhibitor Cocktail Set III (3 µl, Merck, Darmstadt, Germany). Extracts equivalent to 30 µg of total protein were then subjected SDS-PAGE and transferred to nitrocellulose membranes following standard protocols. Primary antibodies against TREM-1 (sc-293450), DAP12 (sc-166084) and GAPDH (ab-9485) were used (Santa Cruz Biotechnology, Santa Cruz, CA, USA; Abcam, Cambridge, Great Britain) for subsequent immunostaining, followed by visualisation with appropriate horseradish peroxidase-coupled secondary antibodies (Santa Cruz Biotechnology) and enhanced chemo-luminescence (34078; Thermo Scientific, Waltham, MA, USA) for detection.

### Statistical analysis

All experiments were performed with at least three biological replicates. Data are presented as mean ± SD. GraphPad Prism 6 (Statcon, Witzenhausen, Germany) was used for statistical data processing with analyses of multiple groups by one-way ANOVA with Dunnett’s multiple comparisons test or ordinary two-way ANOVA being performed as appropriate. All results are statistically significant within *p* < 0.05 unless stated otherwise.

## Electronic supplementary material

Below is the link to the electronic supplementary material.

Supplementary material 1 (DOCX 1030 kb)

## Data Availability

Raw and processed data files are deposited in the Gene Expression Omnibus (GEO) data repository GSE98877 Super Series upon publication.
