# Supplementary material for: Microbially competent 3D skin: a test system that reveals insight into host–microbe interactions and their potential toxicological impact
Source: Arch Toxicol. 2020 Jul 17;94(10):3487–502. doi: 10.1007/s00204-020-02841-z (PMC7502063; doi:10.1007/s00204-020-02841-z)
Supplement: Supplementary file 1 — Supplementary material 1 (DOCX 1030 kb) [file 204_2020_2841_MOESM1_ESM.docx]

**Supplementary Material**

**Method S1**

*Bacterial quantification*

For strain specific quantitative PCR (ss-qPCR) total genomic DNA was isolated from the remaining solution using the RTP® Bacteria DNA Mini Kit (Invitek, STRATEC Molecular GmbH, Berlin, Germany). Prior to ss-qPCR, purity of DNA was estimated using a Nanodrop-1000 Spectrophotometer (Thermo Fisher Scientific, Darmstadt, Germany) and the amount obtained quantified by fluorometry (QuBit 4 Fluorometer, Thermo Fisher Scientific, Darmstadt, Germany).

Primer design

Strain-specific primers were designed for both strains using the WGS data. Sequence alignments and primer design were carried out using the Primer Express (version 3.0.1; Applied Biosystems, Foster City, CA, USA) and *in silico* primer specificity by BLAST in conjunctin with the Accelrys (version 2.5; San Diego, California). The primers selected for further use were 5’- CAT GGC ACC AGC GAA ACC -3’ (forward) and 5’- CTG CTA CTA CCC CTA CTG ATA TTT TCT CT -3’ (reverse) for *M. luteus* and 5’- GTA TCC GCA CCG TCT ACA CC -3’ (forward) and 5’- CAA TGC CTC CAG ATC CAC CA -3’ (reverse) for *P. oleovorans*, targeting a 90 bp intergenic region and a 110 bp region of a 2‑acylglycerophosphoethanolamine acyltransferase, respectively.

Assessment of assay specificity and efficiency

Specificity of both primer pairs was assessed by performing end point PCR with DNA obtained from pure cultures of *M. luteus* and *P. oleovorans* as well as by analysis of the melting curve. Standard curves were generated for both bacterial species using serial dilutions of genomic DNA containing 2 x 10^1^ - 2 x 10^6^ copies of the target gene per reaction. Quantitative (q)PCR was performed on a CFX96 Touch™ Real-Time PCR Detection System (Bio-Rad Laboratories, Hercules, CA, USA) using the SsoAdvanced™ Universal SYBR® Green mix (Bio-Rad Laboratories, Munich, Germany). Amplification reactions were performed in technical triplicates using 5 µl of template DNA in 25 μL of reaction volume with Biorad Hard-Shell^®^ 96-Well PCR plates and 80 nM of forward and reverse primers, respectively (initial denaturation at 95 °C for 5 min, followed by 40 cycles of 95 °C for 15 s and 61 °C for 30 s and an incremental melting curve from 50 °C to 95 °C at 0.5 °C/5 s).

Linear relationship between quantification cycle (C_q_) and log input DNA copy number was evaluated based on three biological replicates and amplification efficiencies (E) calculated based on the slope of standard curve (E=10^−1/slope^−1).

Ss-qPCR

The corresponding amount of *M. luteus* and *P. oleovorans* was subsequently tested for each sample in technical replicates on a 96 well plate. Genomic DNA containing 2 x 10^6^ copies of the target gene served as internal quantification control. Negative controls containing water instead of template were included in each run. Running conditions resembled those used for the generation of standard curves. A reaction was considered positive if the exponential amplification curve exceeded the threshold within 40 cycles. The quantification cycle (C_q_) was then defined as the cycle number at which the sample fluorescence crossed the threshold. Genome copy numbers were calculated based on representative standard curves for each sample.

| **Primer** | **Sequence forward** | **Sequence reverse** |
| --- | --- | --- |
| **CYP1A1** | 5’-TCC AAG AGT CCA CCC TTC C-3’ | 5’-AAG CAT GAT CAG TGT AGG GAT CT-3’ |
| **CYP2D6** | 5`-AAG AAG TCG CTG GAG CAG TG-3’ | 5`-TTG TCC AAG AGA CCG TTG GG-3’ |
| **CYP1B1** | 5`-TGG ATT TGG AGA ACG TAC CG-3’ | 5`-CCA CGA CCT GAT CCA ATT CT-3’ |
| **GAPDH** | 5`-CTC TGC TCC TCC TGT TCG AC-3’ | 5`-ACG ACC AAA TCC GTT GAC TC-3’ |

**Supplementary Table S2.** Primers used for quantitative RT-PCR.

**
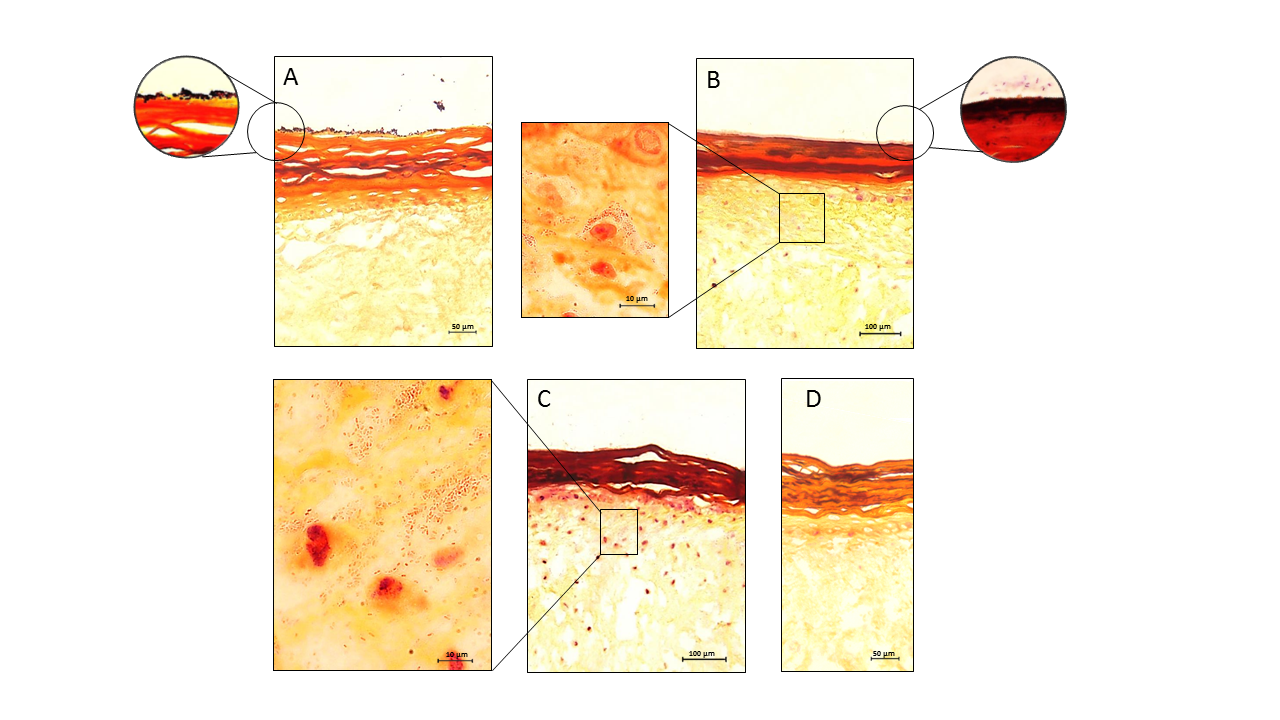
**

**Supplementary Figure S3.** Gram staining of tissue sections of models colonised (A) with *M. luteus*, (B) *P. oleovorans*, (C) a mixture thereof and an untreated control (D). All samples were collected at day 8.


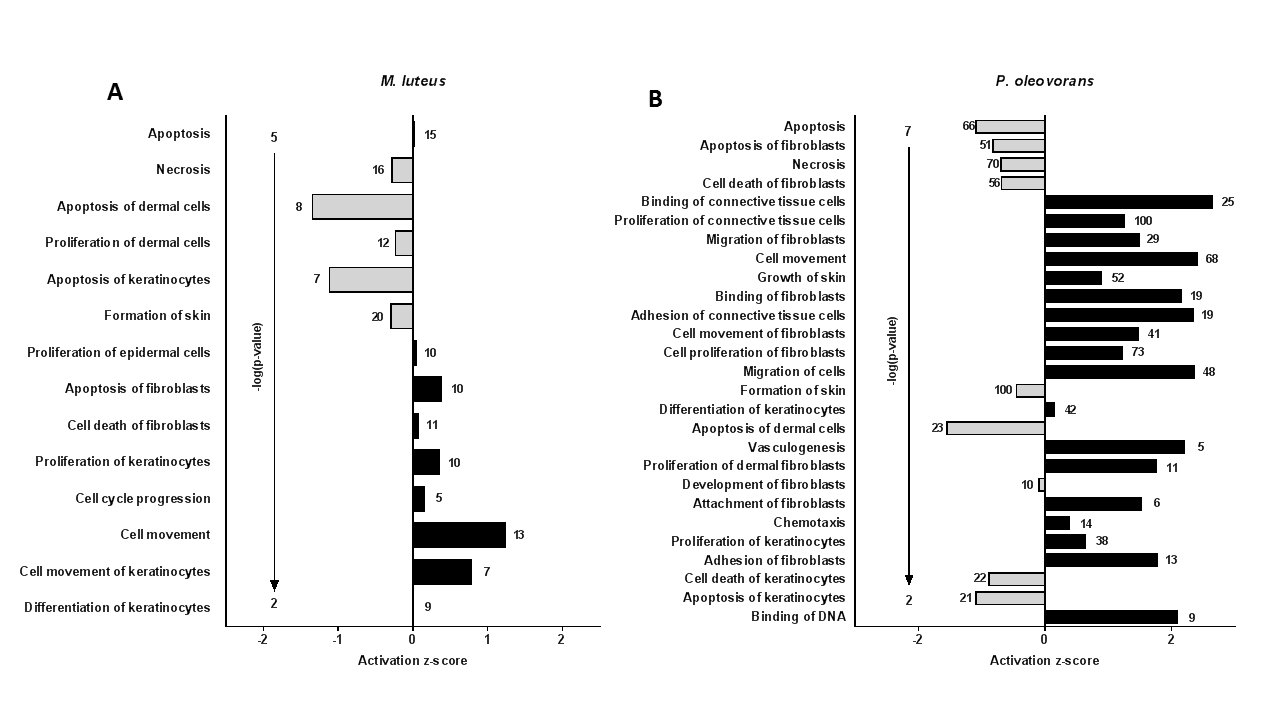


**Supplementary Figure S4.** Functional IPA-analysis of skin models colonised with (A) *M. luteus* and (B) *P. oleovorans*. The activation Z-score indicates an increase or decrease of the corresponding transcriptional pools, with the number of affected transcripts quoted next to each bar.


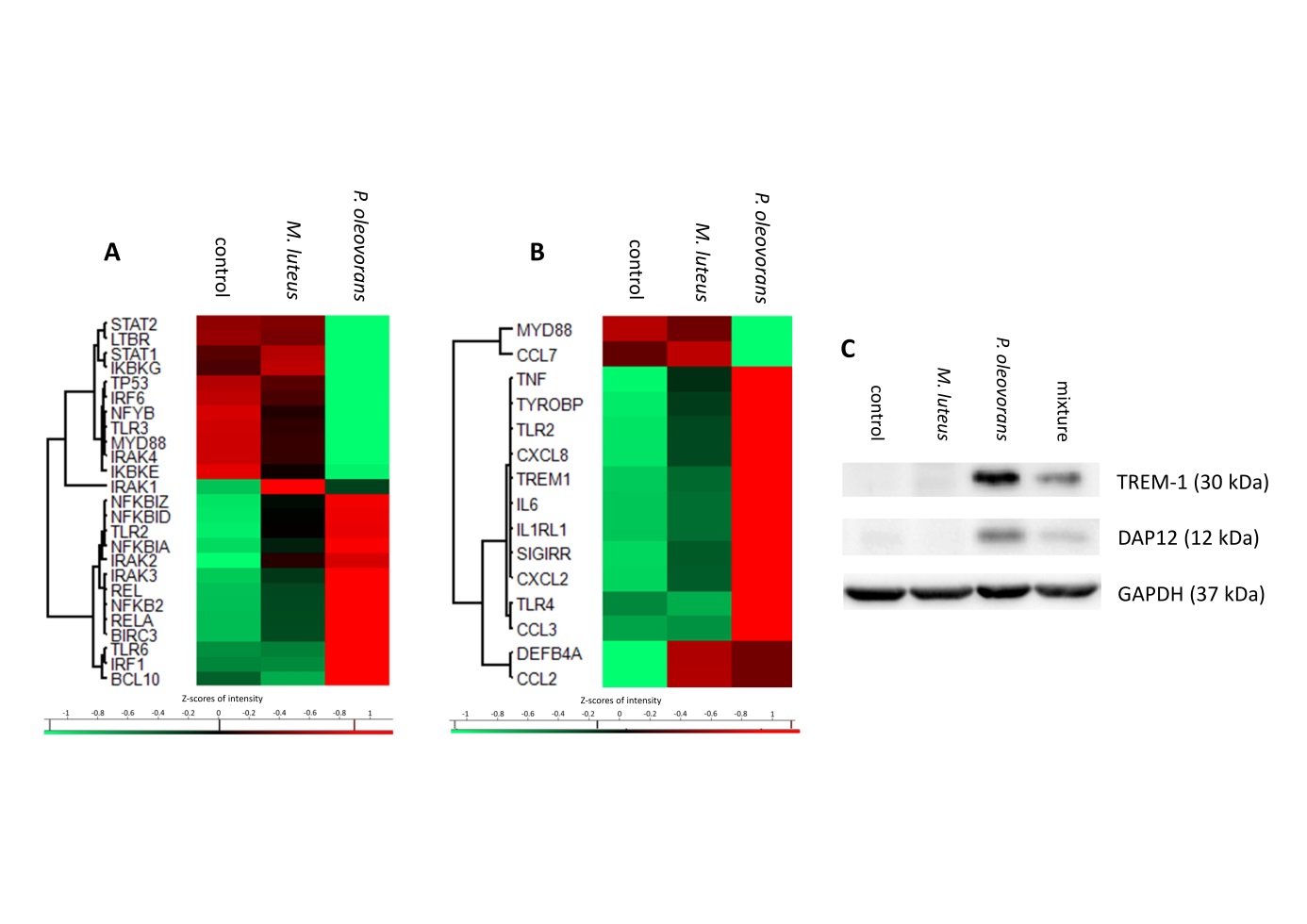


**Supplementary Figure S5.** Transcriptional cluster maps of the NFκB pathway (A) and TREM-signalling related genes (B) in microbially competent skin models at day 8 of microbial colonisation. Shown are the gene symbols and Z-scores of significantly differentially expressed genes with an F-value < 0.05 across three independent experiments. (C) Exemplary shown is an immunoblot of total protein of microbially competent skin models, colonised as indicated.

|  | ***P. oleovorans*** | | ***M. luteus*** | |
| --- | --- | --- | --- | --- |
| **Gene Symbol** | **Fold Change** | **P-value** | **Fold Change** | **P-value** |
| AC171558.1 | 1.79 | 0.0069 | 1.28 | 0.2808 |
| OMP | 2.01 | 0.0064 | 1.01 | 0.9862 |
| OR1A1 | 2.01 | 0.0430 | 1.27 | 0.1219 |
| OR1E1 | 1.45 | 0.0324 | 1.07 | 0.9323 |
| OR1F1 | 2.16 | 0.0269 | 1.36 | 0.3600 |
| OR1J4 | 2.79 | 0.0011 | 2.03 | 0.0428 |
| OR1N1 | 1.7 | 0.0040 | 1.32 | 0.0523 |
| OR1N2 | 1.76 | 0.0036 | 1.35 | 0.1463 |
| OR1S1 | 2.02 | 0.0423 | 1.25 | 0.6662 |
| OR2A7; ARHGEF34P | -1.58 | 0.0209 | -1.37 | 0.1797 |
| OR2A25 | 1.68 | 0.0312 | 1.09 | 0.7259 |
| OR2AP1 | 1.79 | 0.0141 | 1.4 | 0.1018 |
| OR2J2 | 2.42 | 0.0048 | 1.02 | 0.8699 |
| OR2M3 | 1.73 | 0.0376 | 1.3 | 0.7766 |
| OR2M4 | -1.65 | 0.0422 | -2.22 | 0.0178 |
| OR2T2 | 1.84 | 0.0086 | 1.23 | 0.8239 |
| OR2T4 | 1.87 | 0.0076 | -1.11 | 0.7499 |
| OR2T27 | 2.19 | 0.0046 | 1.18 | 0.6003 |
| OR2T35 | 1.71 | 0.0227 | -1.07 | 0.7544 |
| OR2V2 | 2.2 | 0.0093 | 1.3 | 0.2170 |
| OR2Y1 | 2.04 | 0.0089 | 1.93 | 0.0898 |
| OR3A4P; OR3A5P | 1.85 | 0.0042 | 1.46 | 0.0552 |
| OR4A16 | 1.71 | 0.0483 | 1.06 | 0.9652 |
| OR4C3 | 2.68 | 0.0010 | 1.45 | 0.2553 |
| OR4C45 | 1.73 | 0.0433 | 1.11 | 0.6251 |
| OR4D2 | 2.23 | 0.0102 | 1.37 | 0.2484 |
| OR4E2 | 1.78 | 0.0488 | 1.19 | 0.7440 |
| OR4F3 | 1.12 | 0.2879 | -1.75 | 0.0158 |
| OR4F5 | 1.69 | 0.0190 | -1.03 | 0.9798 |
| OR4F15 | 1.85 | 0.0356 | 2.23 | 0.0979 |
| OR4K15 | 1.16 | 0.6018 | -1.31 | 0.0398 |
| OR4M2 | 1.08 | 0.4247 | 1.21 | 0.0474 |
| OR4M2; AC171558.2 | 1.69 | 0.0340 | 1.29 | 0.2340 |
| OR4P4 | 2.39 | 0.0039 | 1.07 | 0.8226 |
| OR4Q3 | 1.88 | 0.0016 | 1.09 | 0.5529 |
| OR4X1 | 1.42 | 0.0404 | -1.05 | 0.9860 |
| OR5AK2 | 1.82 | 0.0408 | -1.19 | 0.6512 |
| OR5AP2 | 1.75 | 0.0258 | 1.33 | 0.2311 |
| OR5AS1 | 1.46 | 0.0164 | 1.04 | 0.6689 |
| OR5H1 | 1.56 | 0.0268 | 1.17 | 0.3041 |
| OR5H6 | 1.72 | 0.0280 | 2.06 | 0.0264 |
| OR5M1 | 2.01 | 0.0037 | -1.02 | 0.9225 |
| OR5M8 | 2.04 | 0.0266 | 1.3 | 0.1823 |
| OR5M9 | 1.7 | 0.0064 | 1.1 | 0.5617 |
| OR5P2 | -15.8 | 3.74E-05 | -2.41 | 0.0324 |
| OR5P2 | -5.84 | 0.0005 | -3.27 | 0.0114 |
| OR5P3 | -2.01 | 0.0061 | -2.02 | 0.0203 |
| OR5P3 | -4.15 | 0.0012 | -1.68 | 0.1121 |
| OR5R1 | 1.15 | 0.1591 | -1.39 | 0.0376 |
| OR6C3 | 2.5 | 0.0207 | 1.73 | 0.2329 |
| OR6C70 | 1.37 | 0.0417 | 1.3 | 0.1876 |
| OR6K2 | 3.28 | 0.0098 | 1.08 | 0.5145 |
| OR6K6 | -1.25 | 0.3533 | -1.38 | 0.0405 |
| OR6M1 | 1.56 | 0.0226 | 1.03 | 0.7031 |
| OR6Q1 | 3.35 | 0.0008 | 1.18 | 0.4992 |
| OR6X1 | 2.31 | 0.0008 | 1 | 0.9155 |
| OR7A5 | 1.95 | 0.0044 | 1.04 | 0.7636 |
| OR7E24 | 2.97 | 0.0018 | 1.56 | 0.3432 |
| OR7G1 | 1.98 | 0.0011 | 1.32 | 0.1336 |
| OR8B3 | -1.51 | 0.3317 | -2.07 | 0.0181 |
| OR8B12 | 1.8 | 0.0098 | 1.72 | 0.1877 |
| OR8S1 | 1.78 | 0.0098 | 1.18 | 0.2960 |
| OR9A2 | 2.04 | 0.0204 | 1.19 | 0.3365 |
| OR9G4 | 1.56 | 0.0263 | -1.01 | 0.3899 |
| OR9I1 | 1.99 | 0.0101 | 1.14 | 0.5733 |
| OR9Q1 | 4.89 | 0.0002 | -1.2 | 0.7552 |
| OR9Q2 | 5.09 | 1.37E-05 | -1.58 | 0.0379 |
| OR10AC1 | 1.76 | 0.0034 | -1.09 | 0.7708 |
| OR10C1 | 1.9 | 0.0087 | 1.14 | 0.5509 |
| OR10J4 | 1.84 | 0.0145 | 1.23 | 0.1774 |
| OR10S1 | 1.61 | 0.0212 | 1.13 | 0.4626 |
| OR12D1 | 1.85 | 0.0085 | 1.19 | 0.6590 |
| OR13A1 | 1.69 | 0.0344 | -1.02 | 0.7281 |
| OR14C36 | 1.5 | 0.0111 | -1.58 | 0.3706 |
| OR51E2 | 1.52 | 0.0332 | -1.03 | 0.9894 |
| OR51F1 | 1.51 | 0.0221 | -1 | 0.6906 |
| OR51H1 | 2.28 | 0.0081 | 1.15 | 0.4326 |
| OR51L1 | 1.61 | 0.0348 | 1.9 | 0.1414 |
| OR52E1 | -2.08 | 0.0386 | -1.6 | 0.0463 |
| OR52E2 | 1.94 | 0.0044 | 1.08 | 0.7729 |
| OR52H1 | 1.65 | 0.0644 | 1.63 | 0.0200 |
| OR52N5 | 1.66 | 0.0223 | 1.23 | 0.2946 |
| OR52R1 | 1.61 | 0.0265 | 1.25 | 0.1126 |
| OR56A1 | 1.83 | 0.0389 | 1.99 | 0.0909 |

**Supplementary Figure S6**. List of olfactory receptors for which co-colonisation led to altered gene expression. Shown are fold changes relative to the control and the respective p-values. Upregulated genes are highlighted in red and downregulated genes in green (p< 0.05).

**
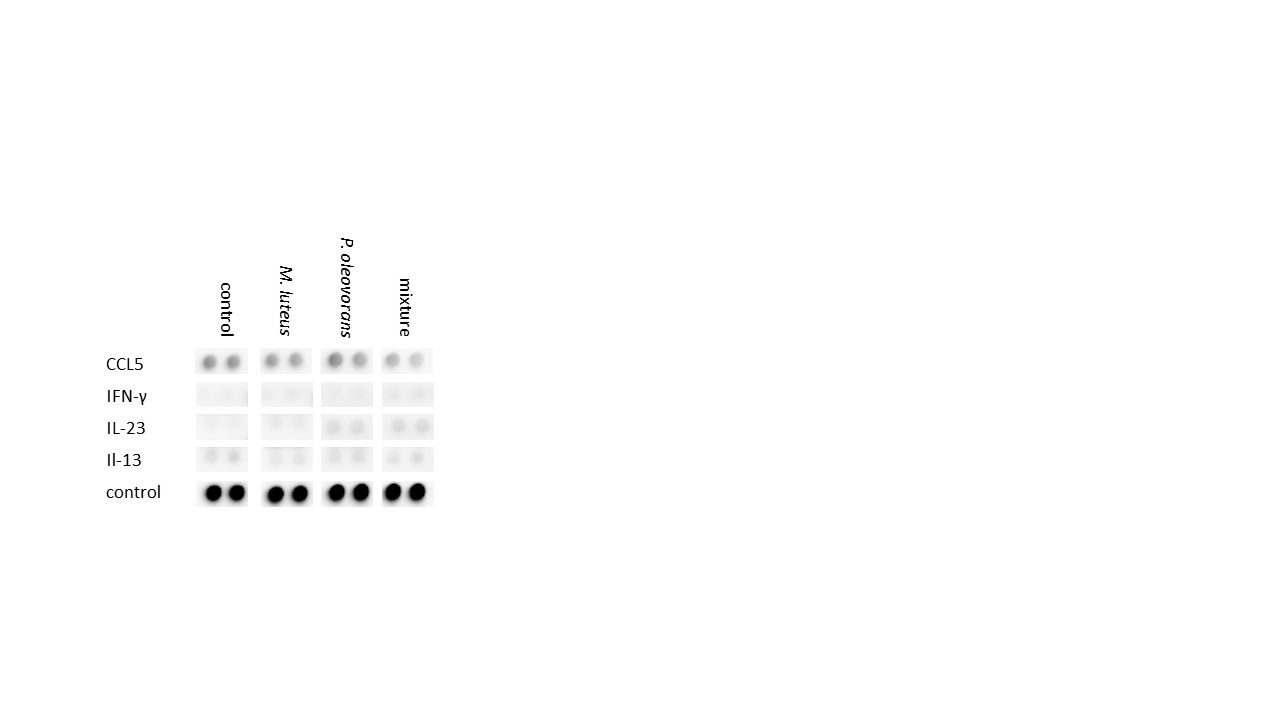
**

**Supplementary Figure S7.** Cytokine excretion of THP-1 cells treated with day 8-supernatant from microbially competent skin, colonised as indicated.
